# Donor‐Derived T‐Cell Redirection With Epcoritamab Achieving Complete Response After Early Post‐Allo‐HSCT Relapse of DLBCL

**DOI:** 10.1002/jha2.70186

**Published:** 2025-11-24

**Authors:** Yoshikazu Ikoma, Yuto Kaneda, Ryoma Shimazu, Daisuke Okamoto, Takuro Matsumoto, Nobuhiko Nakamura, Hiroshi Nakamura, Nobuhiro Kanemura, Hideko Goto, Naoki Katsumura, Masahito Shimizu

**Affiliations:** ^1^ Department of Hematology and Infectious Disease Gifu University Hospital Gifu Japan; ^2^ Center for Nutrition Support and Infection Control Gifu University Hospital Gifu Japan; ^3^ Department of Hematology Gifu Municipal Hospital Gifu Japan; ^4^ Department of Hematology Chuno Kosei Hospital Gifu Japan; ^5^ Department of Gastroenterology Chuno Kosei Hospital Gifu Japan

**Keywords:** bispecific antibodies, diffuse large B‐cell lymphoma, graft‐versus‐host disease, haematopoietic stem cell transplantation, immunotherapy

## Abstract

**Background:**

Epcoritamab, a bispecific CD3×CD20 antibody, offers a promising treatment for relapsed diffuse large B‐cell lymphoma (DLBCL). Its efficacy and safety after allogeneic hematopoietic stem cell transplantation (allo‐HSCT) remain unclear.

**Case Report:**

In a 64‐year‐old man with relapsed DLBCL, epcoritamab showed limited efficacy, achieving stable disease prior to allo‐HSCT; nevertheless, re‐administration after donor T‐cell engraftment resulted in a complete response without severe graft‐versus‐host disease or cytokine release syndrome.

**Conclusions:**

This observation suggests that donor‐derived T‐cell redirection with epcoritamab may enhance antitumor efficacy while maintaining safety in the post‐transplant setting, supporting its potential as a salvage therapy for relapsed DLBCL post‐allo‐HSCT.

## 1 Introduction

Diffuse large B‐cell lymphoma (DLBCL) is the most common aggressive B‐cell malignancy, and relapse or refractory disease remains a major therapeutic challenge [1]. Bispecific T‐cell‐engaging antibodies, exemplified by epcoritamab—a CD3×CD20 construct—have expanded treatment options for heavily pretreated relapsed/refractory DLBCL and achieved durable responses, even after the failure of chimeric antigen receptor T‐cell (CAR‐T) therapy [2, 3]. However, allogeneic haematopoietic stem cell transplantation (allo‐HSCT) remains one of the few potentially curative options for chemorefractory disease or patients who relapse, particularly early after CAR‐T therapy, or do not qualify for further cellular therapy [4]. Early relapse after allo‐HSCT is associated with poor outcomes, and effective salvage options in this setting have not been well defined.

Although immunotherapies such as programmed cell death Protein 1 (PD‐1) inhibitors have been used post‐transplant, they carry a significant risk of severe graft‐versus‐host disease (GVHD) [5, 6]. By contrast, preclinical studies suggest that CD3×CD20 bispecific antibodies may selectively activate T cells within the tumour microenvironment, potentially reducing systemic immune toxicity [7, 8]. Nonetheless, the safety and efficacy of administering epcoritamab soon after allo‐HSCT, when donor‐derived T cells predominate and the risk of immune complications remains high, are largely uncharacterized.

## 2 Case Presentation

Here, we report a patient with early post‐allo‐HSCT relapse of DLBCL who achieved a complete response after re‐administration of epcoritamab, despite having only stable disease prior to transplantation. Notably, no acute or chronic GVHD was observed following treatment, suggesting the hypothesis that redirecting donor‐derived T cells with a bispecific antibody can yield effective tumour control without exacerbating alloreactivity. The present case addresses two key clinical questions: whether epcoritamab can enhance anti‐tumour efficacy in regard to donor T cells after transplantation, and whether this approach is safe in terms of GVHD risk during the early post‐transplant period.

**FIGURE 1:**
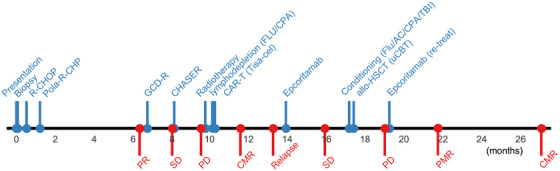
Timeline showing clinical interventions and disease responses. Vertical lines and circles indicate timing of clinical intervention (top, blue) and treatment response (bottom, red). The *x*‐axis shows time since presentation (Month 0). AC, cytarabine; allo‐HSCT, allogeneic haematopoietic stem cell transplantation; CPA, cyclophosphamide; CHASER, cyclophosphamide, cytarabine, etoposide, dexamethasone and rituximab; CMR, complete metabolic response; FLU, fludarabine; GCD‐R, gemcitabine, carboplatin, dexamethasone and rituximab; PD, progressive disease; PMR, partial metabolic response; Pola‐R‐CHP, polatuzumab vedotin, rituximab, cyclophosphamide, doxorubicin and prednisone; PR, partial metabolic response; R‐CHOP, rituximab, cyclophosphamide, doxorubicin, vincristine and prednisone; SD, stable disease; TBI, total body irradiation; Tisa‐cel, tisagenlecleucel; uCBT, umbilical cord blood transplantation.

In 2023, a 64‐year‐old man presented with painless cervical lymphadenopathy and abdominal distension. Positron emission tomography–computed tomography (PET–CT) revealed lymphadenopathy in the cervical, mediastinal, abdominal and inguinal regions. Excisional biopsy confirmed DLBCL of the non‐germinal centre B‐cell‐like subtype according to the Hans algorithm [9]. Baseline laboratory studies showed a lactate dehydrogenase level of 756 U/L and a soluble interleukin‐2 receptor value of 14,022 U/mL. Bone marrow involvement was present. As a result, his disease was classified as Ann Arbor stage IVA [10] with an International Prognostic Index of 3 (indicating high‐intermediate risk) [11]. A timeline of the entire clinical course is shown in Figure 1.

Owing to the aggressive disease progression, accompanied by a suspected diagnosis of B‐cell lymphoma, frontline treatment was initiated with one cycle of rituximab, cyclophosphamide, doxorubicin, vincristine and prednisone. After histopathological confirmation of DLBCL, therapy was switched to five cycles of polatuzumab vedotin, rituximab, cyclophosphamide, doxorubicin and prednisone [12], plus two additional doses of rituximab, resulting in a partial metabolic response on PET–CT [13]. Subsequent salvage therapy with two cycles of gemcitabine, carboplatin, dexamethasone and rituximab [14, 15] led to stable disease, while one cycle of cyclophosphamide, cytarabine, etoposide, dexamethasone and rituximab [16] resulted in disease progression. Bridging radiotherapy (16 Gy) was followed by lymphodepletion (fludarabine and cyclophosphamide) and tisagenlecleucel infusion, which achieved a transient complete metabolic response before relapse 2 months later [13].

**FIGURE 2:**
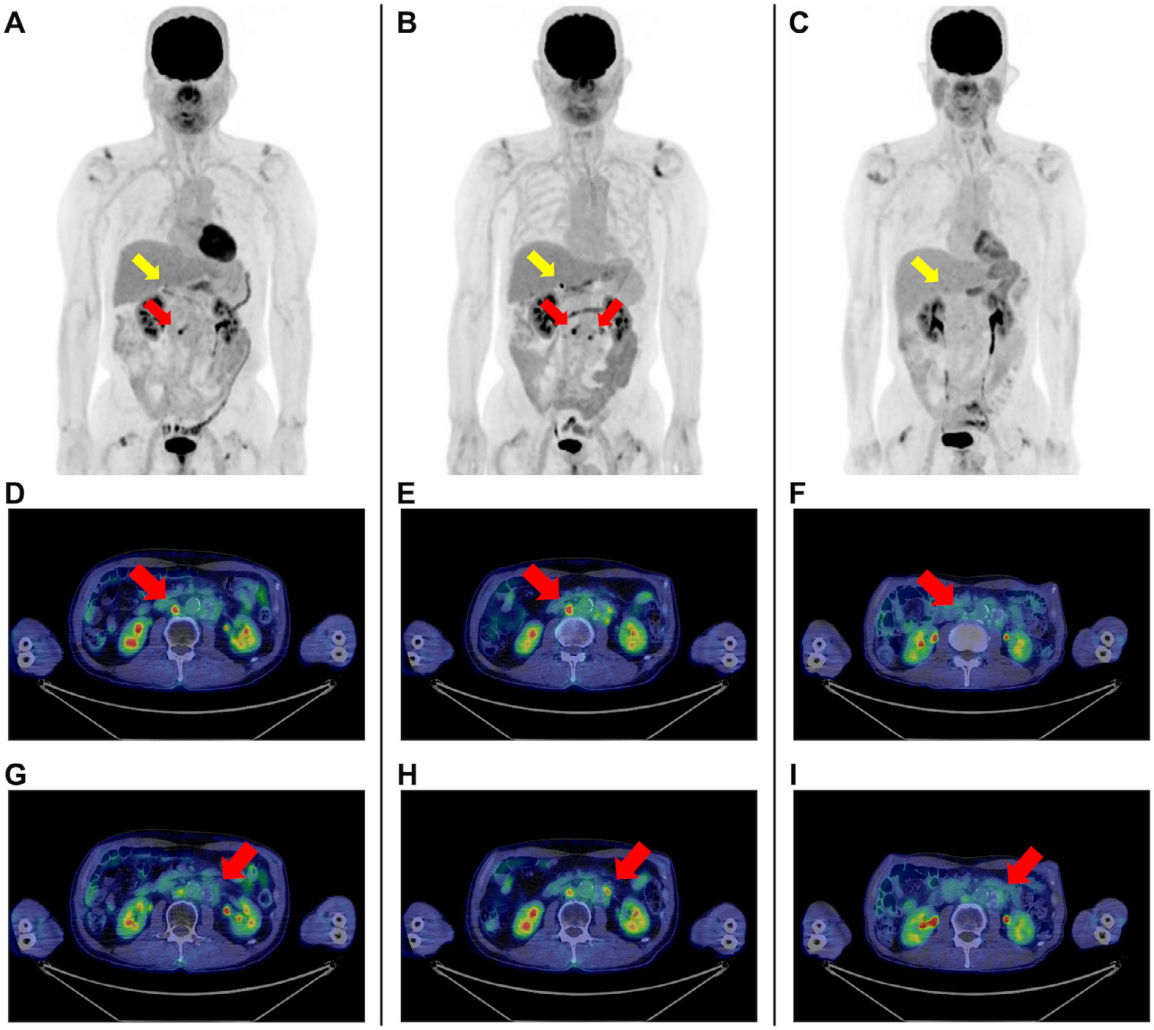
Positron emission tomography–computed tomography (PET–CT) during clinical course. (A–C) Whole‐body maximum intensity projection (MIP) images. (D–I) Axial PET–CT images of the right (D–F) and left (G–I) para‐aortic lymph nodes. Red arrows indicate the para‐aortic lymph nodes of interest, and yellow arrows indicate the right adrenal gland. (A, D, G) After two cycles of epcoritamab (pre‐allogeneic haematopoietic stem cell transplantation [allo‐HSCT]), residual fluorodeoxyglucose (FDG) uptake was observed in the right para‐aortic lymph node (D, maximum standardized uptake value [SUVmax] 7.9), with no significant uptake in the left (G, SUVmax 2.5). Mild physiologic uptake was noted in the right adrenal gland (SUVmax 4.2). (B, E, H) On Day +49 post‐allo‐HSCT, persistent FDG uptake was observed in the right para‐aortic lymph node (E, SUVmax 8.1), with newly appearing uptake in the left (H, SUVmax 5.0) and in the right adrenal gland (SUVmax 11.4), indicating progressive disease. (C, F, I) After nine cycles of epcoritamab post‐allo‐HSCT relapse, complete metabolic response (F, SUVmax 3.0; I, SUVmax 2.4). Mild physiologic uptake remained in the right adrenal gland (SUVmax 3.9).

Epcoritamab, administered with step‐up dosing to 48 mg, was initiated as salvage therapy. The patient developed Grade 1 cytokine release syndrome (CRS) after the first cycle, which resolved following tocilizumab administration. After two cycles, the best response was stable disease (Figure 2A,D,G) [13].

Due to refractory disease, after a total of three cycles of epcoritamab, a single‐unit cord blood allo‐HSCT was performed with non‐myeloablative conditioning (fludarabine, cytarabine, cyclophosphamide and total body irradiation 4 Gy). Neutrophil engraftment was achieved by Day +29, followed by platelet engraftment soon after. A pre‐engraftment immune reaction was transient. No acute or chronic GVHD developed. Full donor chimerism in whole blood and CD3+ cells was achieved by 1 month post‐transplant.

On Day +49 post‐transplant, PET–CT showed persistent fluorodeoxyglucose uptake in the right para‐aortic lymph node and newly appearing uptake in the left para‐aortic lymph node and right adrenal gland (Figure 2B,E,H), indicating progressive disease. Epcoritamab was restarted on Day +57 using the same step‐up schedule. No CRS or GVHD occurred. BK virus haemorrhagic cystitis and cytomegalovirus reactivation developed, but both were controlled with valganciclovir and supportive care. Immunosuppression (cyclosporine) was tapered and discontinued by 4 months post‐transplant, without any GVHD flare.

PET–CT after nine cycles of epcoritamab post‐transplant demonstrated complete metabolic response (Figure 2C,F,I) [13] with sustained full donor chimerism and no GVHD. Monthly maintenance epcoritamab continues without further immune toxicity.

## 3 Discussion

This case highlights two key points regarding the use of epcoritamab for early relapse of DLBCL after allogeneic cord blood transplantation. First, after allo‐HSCT, the patient's T cells were replaced by donor‐derived cells, and re‐administration of epcoritamab led to a complete response, whereas only stable disease was achieved before transplantation when the T cells were autologous. Second, this approach did not cause or worsen GVHD, which suggests that bispecific antibodies can be both effective and safe after transplantation.

The first point centres on the activity of epcoritamab with donor‐derived T cells. The present patient received two cycles of epcoritamab before transplantation, resulting only in stable disease when T cells were autologous. Recent studies have shown that heavily pretreated patients often exhibit impaired T‐cell fitness, which reduces the efficacy of both CAR‐T therapy and bispecific antibodies [17, 18]. Thus, the lack of response to epcoritamab before allo‐HSCT in the present patient may have been influenced by this compromised T‐cell fitness. However, after transplantation replaced the T‐cell compartment with donor‐derived cells, a second course of epcoritamab led to a complete response. This improvement likely reflects the restored fitness and increased functional capacity of donor‐derived T cells after transplant, enabling more effective redirection by the bispecific antibody. Although cord blood transplantation is generally associated with delayed engraftment and slow T‐cell recovery, donor‐type immune reconstitution was confirmed in this case. Chimerism analysis on Day +19 after transplantation demonstrated complete donor‐type CD3⁺ cells, and flow cytometry showed gradual T‐cell recovery with CD3⁺ T‐cell counts of 91 cells/µL at approximately 2.5 months post‐transplant and 173 cells/µL at 6 months. These findings suggest that sufficient donor‐derived T‐cell reconstitution had already begun at the time of epcoritamab re‐treatment. Prior reports of bispecific antibody use, including epcoritamab, in the early post‐allo‐HSCT setting are extremely limited. To our knowledge, no previous case has described a complete response without significant GVHD in this context. Possible mechanisms include synergy between the graft‐versus‐lymphoma effect and T‐cell redirection, as well as increased T‐cell receptor diversity after transplant, which could enhance immune surveillance and antitumor activity [2, 4]. Recent studies also suggest that donor T cells can recognize a broader set of lymphoma antigens than autologous cells, which may partly explain this favourable response. While a delayed effect of prior CAR‐T therapy cannot be fully excluded, the relapse occurred within 2 months after tisagenlecleucel infusion. Given the complete donor‐type CD3⁺ chimerism and evidence of T‐cell recovery, persistence of CAR‐T cells was unlikely, making a delayed or synergistic contribution from prior tisagenlecleucel therapy less likely.

The second point is the observed safety and clinical applicability of this strategy. Despite starting immunotherapy soon after transplantation, the present patient did not develop acute or chronic GVHD, and pre‐engraftment immune reactions were well controlled. By contrast, when used after allogeneic transplantation, immune checkpoint inhibitors are associated with a high risk of severe GVHD [5, 6]. For bispecific antibodies such as epcoritamab, preclinical work shows that CD3×CD20 constructs form focused immune synapses and confine T‐cell activation to the tumour interface, resulting in lower systemic interleukin‐2 release and reduced off‐tumour toxicity [7, 8]. Consistent with this concept, a recent series of eight patients who underwent allo‐HSCT after prior CD3×CD20 bispecific antibodies reported manageable acute and chronic GVHD, although infection‐related mortality remained high [19]. Blinatumomab, a bispecific CD3×CD19 antibody, has been successfully used after allo‐HSCT in acute lymphoblastic leukaemia, with recent studies reporting a low risk of severe GVHD [20]. These findings support the feasibility and safety of bispecific antibodies in the post‐transplant setting, provided T‐cell fitness and immune‐mediated toxicity are adequately managed. While bispecific antibody therapy does not appear to exacerbate GVHD, careful monitoring for infectious complications is essential in the peri‐transplant period. Therefore, both preclinical and clinical studies support the idea that epcoritamab may carry less risk of systemic immune complications than checkpoint blockade. Critically, no peer‐reviewed clinical studies have described epcoritamab after allo‐HSCT; therefore, the present report provides early evidence that epcoritamab can be administered post‐transplant without provoking GVHD or high‐grade CRS. In the present case, CRS was mild, and other complications, such as cytomegalovirus reactivation and BK virus cystitis, were manageable with standard therapies. Given the absence of formal safety data in this setting, close monitoring for GVHD and CRS remains essential whenever bispecific antibodies are used after allo‐HSCT. We acknowledge that umbilical cord blood transplantation is associated with a lower inherent risk of GVHD compared to peripheral blood or bone marrow sources. Therefore, our findings may not be fully generalizable to other graft types. Furthermore, graft manipulation strategies such as in vivo or ex vivo T‐cell depletion may significantly influence the immune environment and GVHD risk, and should be considered when interpreting the safety of bispecific antibodies in the post‐transplant setting.

Clinically, bispecific antibodies offer an ‘off‐the‐shelf’ salvage therapy for patients who relapse after transplantation, especially those who are not candidates for CAR‐T therapy or donor lymphocyte infusion. This case supports the possibility that bispecific antibodies can provide effective and safe treatment in this challenging setting. At the same time, we recognize that this is a single case, and the findings remain anecdotal and hypothesis‐generating, requiring validation in larger cohorts. Future studies are warranted to determine the optimal timing, dosing, and risk management for these therapies after transplantation.

## 4 Conclusion

The present case indicates that donor‐derived T‐cell redirection by epcoritamab can induce a complete response after the early post‐transplant relapse of DLBCL, and that this therapy does not worsen GVHD. However, careful monitoring and infection control are essential for safe use. As bispecific antibodies become more widely available, future protocols should focus on maximizing their benefit while minimizing complications in posttransplant contexts.

## Author Contributions

Y.I., Y.K., R.S., D.O., T.M., N.N., H.N., N. K., H.G., N. Katsumura and M.S. contributed to the conception of the case. Y.I., Y.K., R.S. and H.G. collected the clinical data. Y.I. and N. Kanemura drafted the manuscript. N. Kanemura and M.S. critically revised the manuscript. All authors approved the final version of the manuscript and are accountable for all aspects of the work.

## Funding

The authors have nothing to report.

## Ethics Statement

This case report complies with the Declaration of Helsinki.

## Consent

Written informed consent was obtained from the patient for publication of this report and all accompanying images.

## Conflicts of Interest

Nobuhiko Nakamura has received honoraria from Chugai Pharmaceutical Co., Ltd., Kyowa Kirin Co., Ltd., AbbVie GK, Janssen Pharmaceutical K.K., Sanofi, Novartis, MSD K.K., Sumitomo Pharma Co., Ltd., Nippon Kayaku Co., Ltd., Nippon Shinyaku Co., Ltd., Meiji Seika Pharma Co., Ltd. and AstraZeneca K.K. The other authors declare no conflicts of interest.

## Data Availability

The data that support the findings of this case report are not publicly available due to patient privacy concerns. Data may be available from the corresponding author upon reasonable request and with appropriate institutional approval.

## References

[1] M. Crump , S. S. Neelapu , U. Farooq , et al., “Outcomes in Refractory Diffuse Large B‐Cell Lymphoma: Results From the International SCHOLAR‐1 Study,” Blood 130, no. 16 (2017): 1800–1808, 10.1182/blood-2017-03-769620. 28774879 PMC5649550

[2] C. Thieblemont , T. Phillips , H. Ghesquieres , et al., “Epcoritamab, a Novel, Subcutaneous CD3xCD20 Bispecific T‐Cell–Engaging Antibody, in Relapsed or Refractory Large B‐Cell Lymphoma: Dose Expansion in a Phase I/II Trial,” Journal of Clinical Oncology 41, no. 12 (2023): 2238–2247, 10.1200/JCO.22.01725. 36548927 PMC10115554

[3] C. Thieblemont , Y. H. Karimi , H. Ghesquieres , et al., “Epcoritamab in Relapsed/Refractory Large B‐Cell Lymphoma: 2‐Year Follow‐Up From the Pivotal EPCORE NHL‐1 Trial,” Leukemia 38, no. 12 (2024): 2653–2662, 10.1038/s41375-024-02410-8. 39322711 PMC11588654

[4] L. Castagna , R. Bono , S. Tringali , et al., “The Place of Allogeneic Stem Cell Transplantation in Aggressive B‐Cell Non‐Hodgkin Lymphoma in the Era of CAR‐T‐Cell Therapy,” Frontiers in Medicine 9 (2022): 1072192, 10.3389/fmed.2022.1072192. 36561713 PMC9763323

[5] B. M. Haverkos , D. Abbott , M. Hamadani , et al., “PD‐1 Blockade for Relapsed Lymphoma Post–Allogeneic Hematopoietic Cell Transplant: High Response Rate but Frequent GVHD,” Blood 130, no. 2 (2017): 221–228, 10.1182/blood-2017-01-761346. 28468799 PMC5510790

[6] A. Ijaz , A. Y. Khan , S. U. Malik , et al., “Significant Risk of Graft‐Versus‐Host Disease With Exposure to Checkpoint Inhibitors Before and After Allogeneic Transplantation,” Biology of Blood and Marrow Transplantation 25, no. 1 (2019): 94–99, 10.1016/j.bbmt.2018.08.028. 30195074 PMC6310648

[7] P. J. Engelberts , I. H. Hiemstra , B. De Jong , et al., “DuoBody‐CD3xCD20 Induces Potent T‐Cell‐Mediated Killing of Malignant B Cells in Preclinical Models and Provides Opportunities for Subcutaneous Dosing,” EBioMedicine 52 (2020): 102625, 10.1016/j.ebiom.2019.102625. 31981978 PMC6992935

[8] A. Leithner , O. Staufer , T. Mitra , et al., “Solution Structure and Synaptic Analyses Reveal Determinants of Bispecific T Cell Engager Potency,” Proceedings of the National Academy of Sciences 122, no. 22 (2025): e2425781122, 10.1073/pnas.2425781122. PMC12146755 40445758

[9] C. P. Hans , “Confirmation of the Molecular Classification of Diffuse Large B‐Cell Lymphoma by Immunohistochemistry Using a Tissue Microarray,” Blood 103, no. 1 (2004): 275–282, 10.1182/blood-2003-05-1545. 14504078

[10] P. P. Carbone , H. S. Kaplan , K. Musshoff , D. W. Smithers , and M. Tubiana , “Report of the Committee on Hodgkin's Disease Staging Classification,” Cancer Research 31, no. 11 (1971): 1860–1861. 5121694

[11] The International Non‐Hodgkin's Lymphoma Prognostic Factors Project , “A Predictive Model for Aggressive Non‐Hodgkin's Lymphoma.” New England Journal of Medicine 329, no. 14 (1993): 987–994, 10.1056/NEJM199309303291402. 8141877

[12] H. Tilly , F. Morschhauser , L. H. Sehn , et al., “Polatuzumab Vedotin in Previously Untreated Diffuse Large B‐Cell Lymphoma,” New England Journal of Medicine 386, no. 4 (2022): 351–363, 10.1056/NEJMoa2115304. 34904799 PMC11702892

[13] B. D. Cheson , R. I. Fisher , S. F. Barrington , et al., “Recommendations for Initial Evaluation, Staging, and Response Assessment of Hodgkin and Non‐Hodgkin Lymphoma: The Lugano Classification,” Journal of Clinical Oncology 32, no. 27 (2014): 3059–3067, 10.1200/JCO.2013.54.8800. 25113753 PMC4979083

[14] A. K. Gopal , O. W. Press , A. R. Shustov , et al., “Efficacy and Safety of Gemcitabine, Carboplatin, Dexamethasone, and Rituximab in Patients With Relapsed/Refractory Lymphoma: A Prospective Multi‐Center Phase II Study by the Puget Sound Oncology Consortium,” Leukemia & Lymphoma 51, no. 8 (2010): 1523–1529, 10.3109/10428194.2010.491137. 20578815 PMC3018339

[15] Y. Ikoma , N. Nakamura , J. Kitagawa , et al., “A Phase II Study of Gemcitabine, Carboplatin, Dexamethasone, and Rituximab in Patients With Relapsed or Refractory Non‐Hodgkin Lymphoma,” Hematological Oncology 42, no. 1 (2024): e3236, 10.1002/hon.3236. 37932900

[16] Y. Oki , M. Ogura , H. Kato , et al., “Phase II Study of a Salvage Regimen Using Cyclophosphamide, High‐Dose Cytarabine, Dexamethasone, Etoposide, and Rituximab in Patients With Relapsed or Refractory B‐Cell Non‐Hodgkin's Lymphoma,” Cancer Science 99, no. 1 (2008): 179–184, 10.1111/j.1349-7006.2007.00662.x. 17991293 PMC11159101

[17] J. A. Fraietta , S. F. Lacey , E. J. Orlando , et al., “Determinants of Response and Resistance to CD19 Chimeric Antigen Receptor (CAR) T Cell Therapy of Chronic Lymphocytic Leukemia,” Nature Medicine 24, no. 5 (2018): 563–571, 10.1038/s41591-018-0010-1. PMC6117613 29713085

[18] A. H. Wei , J.‐M. Ribera , R. A. Larson , et al., “Biomarkers Associated With Blinatumomab Outcomes in Acute Lymphoblastic Leukemia,” Leukemia 35, no. 8 (2021): 2220–2231, 10.1038/s41375-020-01089-x. 33542479 PMC8324476

[19] M. Peña , C. Montané , A. Paviglianiti , et al., “Outcomes of Allogeneic Hematopoietic Cell Transplantation After Bispecific Antibodies in Non‐Hodgkin Lymphomas,” Bone Marrow Transplantation 58, no. 11 (2023): 1282–1285. 37626265 10.1038/s41409-023-02069-2

[20] M. R. Gaballa , P. Banerjee , D. R. Milton , et al., “Blinatumomab Maintenance After Allogeneic Hematopoietic Cell Transplantation for B‐Lineage Acute Lymphoblastic Leukemia,” Blood 139, no. 12 (2022): 1908–1919, 10.1182/blood.2021013290. 34914826 PMC8952188

